# Influence of Sedation Level and Ventilation Status on the Diagnostic Validity of Delirium Screening Tools in the ICU—An International, Prospective, Bi-Center Observational Study (IDeAS)

**DOI:** 10.3390/medicina56080411

**Published:** 2020-08-13

**Authors:** Flavio E. Nacul, Nicolas Paul, Claudia D. Spies, Henriette Sechting, Thomas Hecht, Jörn S. Dullinger, Sophie K. Piper, Alawi Luetz, Felix S. Balzer, Klaus-Dieter Wernecke, Anna Karinina Sa, Carolina Barros Ferreira da Costa, Lisa Eymold, Chokri Chenitir, Björn Weiss

**Affiliations:** 1 Surgical Critical Care Medicine, Hospital Pro-Cardiaco, Rio de Janeiro, RJ 22280-003, Brazil; fnacul@uol.com.br (F.E.N.); annasa@procardiaco.com.br (A.K.S.); carolbfcosta@gmail.com (C.B.F.d.C.); 2 Department of Anesthesiology and Operative Intensive Care Medicine (CCM, CVK), Charité—Universitätsmedizin Berlin, Corporate Member of Freie Universität Berlin, Humboldt-Universität zu Berlin and Berlin Institute of Health, 10117 Berlin, Germany; nicolas.paul@charite.de (N.P.); claudia.spies@charite.de (C.D.S.); henriette.sechting@charite.de (H.S.); tommyhecht@web.de (T.H.); jdullinger@schoen-klinik.de (J.S.D.); alawi.luetz@charite.de (A.L.); felix.balzer@charite.de (F.S.B.); lisa.eymold@charite.de (L.E.); chokrichenitir@web.de (C.C.); 3 Institute of Biometry and Clinical Epidemiology, Charité—Universitätsmedizin Berlin, Corporate Member of Freie Universität Berlin, Humboldt-Universität zu Berlin and Berlin Institute of Health, 10117 Berlin, Germany; sophie.piper@charite.de (S.K.P.); wernecke@sostana.de (K.-D.W.); 4 Berlin Institute of Health (BIH), 10178 Berlin, Germany; 5 Department of Healthcare Management, Technische Universität Berlin, 10623 Berlin, Germany

**Keywords:** delirium, critical illness, intensive care, sedation, ventilation, sensitivity and specificity, test validity

## Abstract

*Background and objectives:* The use of delirium screening instruments (DSIs) is recommended in critical care practice for a timely detection of delirium. We hypothesize that the patient-related factors “level of sedation” and “mechanical ventilation” impact test validity of DSIs. *Materials and Methods:* This is a prospective, bi-center observational study (clinicaltrials.gov: NCT01720914). Critically ill patients were screened for delirium daily for up to seven days after enrollment using the Nursing Delirium Screening Scale (Nu-DESC), Intensive Care Delirium Screening Checklist (ICDSC), and Confusion Assessment Method for the Intensive Care Unit (CAM-ICU). Reference standard for delirium diagnosis was the neuropsychiatric examination using the criteria of the Diagnostic and Statistical Manual of Mental Disorders, Fourth Edition, Text Revision (DSM-IV-TR). Immediately before delirium assessment, ventilation status and sedation levels were documented. *Results:* 160 patients were enrolled and 151 patients went into final analysis. Delirium incidence was 23.2%. Nu-DESC showed a sensitivity and specificity of 88.5%, a positive predictive value (PPV) of 71.9%, and a negative predictive value (NPV) of 95.8%. ICDSC had a sensitivity of 62.5%, a specificity of 92.4%, a PPV of 71.4%, and a NPV of 89.0%. CAM-ICU showed a sensitivity of 75.0%, a specificity of 94.7%, a PPV of 85.7%, and a NPV of 90.0%. For Nu-DESC and ICDSC, test validity was significantly better for non-sedated patients (Richmond Agitation Sedation Scale (RASS) 0/−1), whereas test validity for CAM-ICU in a severity scale version showed no significant differences for different sedation levels. No DSI showed a significant difference in test validity between noninvasively and invasively ventilated patients. *Conclusions:* Test validities of DSIs were comparable to previous studies. The observational scores ICDSC and Nu-DESC showed a significantly better performance in awake and drowsy patients (RASS 0/−1) when compared with other sedation levels. Physicians should refrain from sedation whenever possible to avoid suboptimal performance of DSIs.

## 1. Introduction

ICU delirium is a severe organ dysfunction that affects up to 82% of the critically ill patients [1]. It is associated with an increased cognitive impairment, an increased length of mechanical ventilation, additional time spent in the hospital, as well as an increased mortality [1,2,3]. Due to its clinical relevance, a reliable routine screening for ICU-delirium with a validated score is recommended in clinical practice guidelines [4,5]. Although the reference standard for delirium assessment is the criteria of the Diagnostic and Statistical Manual of Mental Disorders (DSM) by the American Psychiatric Association (APA) [6], daily assessment is recommended with a validated delirium screening instrument (DSI). Examples are the Confusion Assessment Method for the ICU (CAM-ICU) [7], the Intensive Care Delirium Screening Checklist (ICDSC) [8], and the Nursing Delirium Screening Scale (Nu-DESC) [9]. Despite being used in routine delirium screening, the scores have very different psychometric characteristics [10]. Applied by nurses and physicians, the CAM-ICU uses a flowchart to assess the domains acute onset of mental status change or fluctuating course, inattention, disorganized thinking, and altered level of consciousness. In a binary decision, a patient is considered delirious if he is positive in the first and second domain as well as in either the third or fourth domain [7,11]. In contrast, the ICDSC and Nu-DESC allow a differentiation of symptoms and their severity by the use of a one-dimensional scale. The ICDSC entails eight items, namely disorientation, psychomotor alterations, altered level of consciousness, inattention, inappropriate mood or speech, sleep/wake cycle disturbance, fluctuation of symptoms, and hallucination. Each positive item equals one point, and a patient is considered delirious if he or she scores at least four points [8,11]. Further, it has been shown that patients who suffer from delirious symptoms, but do not meet all criteria for delirium, have an impaired outcome, which has been labeled as subsyndromal delirium (SSD) [12]. An ICDSC score of one to three points is considered subsyndromal delirium [11]. Both the CAM-ICU and the ICDSC have been validated for patient populations in a variety of languages, cultural contexts, medical, surgical, as well as mixed medical/surgical ICUs [13]. The observational Nu-DESC comprises five items, each of which is scored from zero to two: inappropriate behavior, psychomotor retardation, inappropriate communication, illusions/hallucinations and disorientation. Patients with a score of at least two are considered delirious [9]. The Nu-DESC has been validated for different languages and for use in surgical ICUs as well as postoperative settings and geriatric wards [14,15]. Unlike the Nu-DESC and ICDSC, the CAM-ICU does not use an ordinal scale. To tackle this disadvantage, a “severity scale” (ss) CAM-ICU was introduced in the pediatric fields, which allows to scale symptoms and report the CAM-ICU result in an ordinal scale as long as it is performed as a worksheet, not as a flowchart [16]. Instead of a binary “yes/no” decision, the ssCAM-ICU uses the operation of rank ordering. The content of the test itself is the same. Other groups used an analogous method which has been validated in the adult context (CAM-ICU-7) [17].

Screening instruments require an implementation and staff training phase [18]. In terms of accessibility, the CAM-ICU, for example, has undergone a translation and validation process in different languages. Nevertheless, it is noticeable that the sensitivity ranges from 72.5% up to nearly 100% [13]. Level of sedation, ventilation status, and staff training have been proposed to cause variances in sensitivities. There has been no systematic approach to assess the influence of the level of sedation and status of ventilation on the validity of DSIs yet. This study aims to investigate the influence of these covariates on DSI validity. We primarily hypothesize that there is a difference in the diagnostic sensitivity and specificity of the DSIs between sedated and nonsedated patients.

## 2. Materials and Methods

### 2.1. Study Design

This is a prospective, bi-center, international observational study, registered at clinicaltrials.gov (NCT01720914). The study was conducted in accordance with the Declaration of Helsinki, and the protocol was approved by the Ethics Committee at Charité-Universitätsmedizin Berlin, Berlin, Germany (EA1/196/12) as well as the Ethics Committee of Hospital Pro-Cardiaco, Rio de Janeiro, Brazil (2013/571). Informed consent followed local regulations: informed consent was waived in Berlin as the assessment of scores is part of a national guideline (AWMF 001-012), and only non-person-related data was analyzed. In Rio de Janeiro, written informed consent by patient or family was obtained.

### 2.2. Study Population

Critically ill patients with at least 18 years of age and an ICU length of stay of at least 24 h were enrolled at Charité-Universitätsmedizin Berlin, Berlin, Germany, and Hospital Pro-Cardiaco, Rio de Janeiro, Brazil. Exclusion criteria were neurosurgical patients, severe brain injury, intracerebral bleeding, stroke, inability to communicate due to anacousia or severe hearing loss, and insufficient language comprehension.

### 2.3. Delirium Assessment Procedure

**Figure 1:**
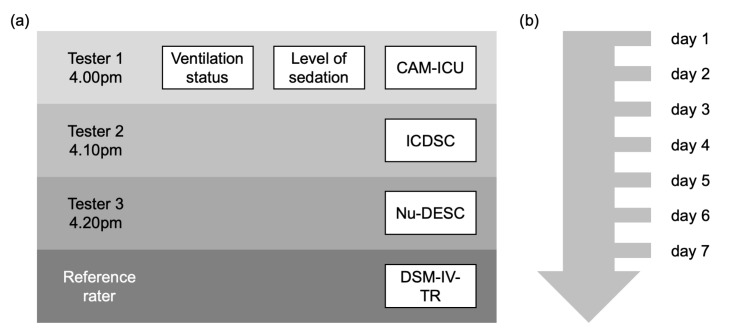
(**a**) Daily delirium screening schedule. The first tester started at 4.00pm, and the last tester finished at about 5.00pm. Ventilation status and level of sedation were assessed with the first delirium screening. (**b**) Patients were assessed daily for up to seven days after study inclusion or until ICU discharge. Abbreviations: CAM-ICU = Confusion Assessment Method for the ICU; ICDSC = Intensive Care Delirium Screening Checklist; Nu-DESC = Nursing Delirium Screening Scale; DSM-IV-TR = Diagnostic and Statistical Manual of Mental Disorders, Fourth Edition, Text Revision.

Every day in the afternoon, enrolled patients were screened for delirium using the Nu-DESC, ICDSC, and CAM-ICU. One assessment tool was applied by one tester (see Figure 1), and testers were blinded to the results of the other testers. Right after assessment, delirium scores were documented with a patient-specific pseudonym in an electronic case report form (eCRF). In case of coma or deep sedation (Richmond Agitation Sedation Scale (RASS) −4 to −5), screening was not conducted on that particular day. There was no explicit hold of sedation prior to the delirium assessment. Aside from the screening in this study, delirium screening was conducted by ICU staff according to standard operating procedures (SOPs). Observation was continued from the time of study inclusion for 7 days or until discharge. The ssCAM-ICU was calculated in an analogous method to that used by Luetz et al. to plot the CAM-ICU values on an ordinal scale [16].

#### 2.3.1. Neuropsychiatric Examination According to DSM-IV-TR

The daily neuropsychiatric examination was performed in line with previous studies using the DSM-IV—Text Revision (TR). The examination was conducted by two reference raters (B.W., J.S.D.) in Berlin and one reference rater (C.B.F.d.C.) in Rio de Janeiro. Reference raters from both centers met in person to ensure an equal approach. The diagnosis was made on the basis of available information from the medical record, medical history, a patient assessment, and interviews with nurses as well as attending physicians. The training of reference raters was led by a board-certified neurologist-intensivist (J.S.D.).

#### 2.3.2. Delirium Screening

The study staff were trained in the application of the scores. At least 20 tests were carried out under supervision and a simulation training was completed. Each score was performed by one specific staff member, which was determined before commencement of the study. Due to staffing reasons, a change in the raters had to take place for Nu-DESC assessments.

### 2.4. Assessment of Covariates and Additional Patient Information

Immediately before daily delirium assessment, sedation and ventilation status were documented. Sedation was measured using the RASS. On the day of enrolment, age, gender, body mass index, height, weight, severity of illness (Acute Physiology and Chronic Health Disease Classification System II (APACHE II), Simplified Acute Physiology Score (SAPS II), Sepsis-related Organ Failure Assessment (SOFA) score), and cause as well as mode of ICU admission were documented.

### 2.5. Sample Size Calculation

We presumed a delirium incidence rate of 40% [14], a delirium test sensitivity of 90% for nonsedated patients (RASS 0/−1), 60% for sedated patients (RASS < −1), 90% for noninvasively ventilated patients, and 70% for invasively ventilated patients. Using the expected rates with smaller difference between groups and adjusting for overall six comparisons (three DSIs with two comparisons each) using the Bonferroni correction α = 0.05/6 = 0.0083, we determined a sample size of at least 128 patients (dropouts not included) for a power of 90% using the Fisher’s exact test of equal proportions (nQuery Advisor^®^ Release 7.0, Stat. Solutions Ltd. & South Bank, Crosse’s Green, Cork, Ireland).

### 2.6. Statistical Analysis

Descriptive statistics of the study population with corresponding distributions are presented as either medians with limits of the interquartile range (25th and 75th percentile) or as absolute (n) or relative (%) frequencies. Differences in characteristics between patients without delirium and patients with at least one episode of delirium were compared using the nonparametric Mann–Whitney U test for continuous variables, Fisher’s exact test for frequencies with two categories, and Chi-squared test for frequencies with more than two categories. Test validity in terms of sensitivity, specificity, and positive and negative predictive values (PPV and NPV) were calculated with respective 95% confidence intervals (CIs). For test validity calculations across all subgroups, the first measurement of the respective DSI and DSM-IV-TR for each patient was considered. McNemar test was used to compare test validity between DSIs. For test validity calculations depending on covariates, measurements were grouped in three subgroups according to RASS levels (RASS < −1; RASS −1 or 0; RASS > 0) or two subgroups according to ventilation status (invasive ventilation (nasopharyngeal tube, oropharyngeal tube, tracheostomy) or no/noninvasive ventilation (mask or no airway)). For analysis, the first measurement of each patient and, if a patient changed subgroups of sedation or ventilation within the study period, the first measurement of the respective subgroup were used. Receiver operating characteristic (ROC) curves were plotted, area under the curve (AUC) was determined, and Youden Index was calculated to determine ideal cut-offs for Nu-DESC, ICDSC, and ssCAM-ICU. AUCs for covariates were compared as described by DeLong et al. [19]. Finally, we fitted a parametric model for the ROC curve, adjusting for covariate effects, using bootstrapping and a probit link between covariates and the ROC curve. Then, we plotted covariate-adjusted ROC curves. In this step, all delirium measurements were included in the analysis. To account for the correlation between the delirium assessments within the same patients on different days (clustered data), each patient was used as resampling unit for the resampling procedure. Analysis was carried out using STATA 13.1 (StataCorp LP, College Station, TX, USA).

## 3. Results

### 3.1. Study Population and Delirium Incidence Rate

**Figure 2:**
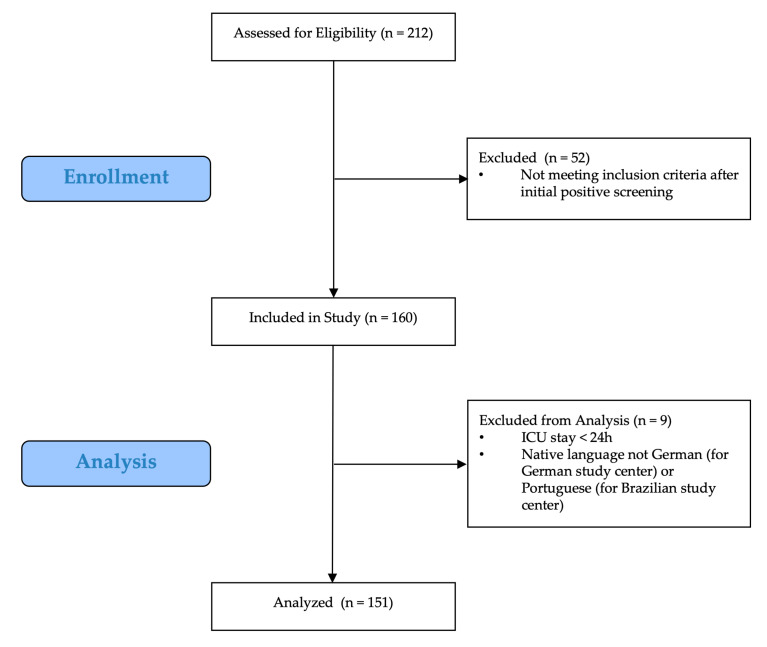
Consort diagram for inclusion of study population.

A total of 160 patients were included in the study (see Figure 2). Nine patients were excluded because their native language was not German (Germany)/Portuguese (Brazil), or ICU stay was less than 24 h, leaving 151 patients for data analysis. 35 patients (23%) were tested positive for delirium according to DSM-IV-TR at least once (see Table 1). Patients with delirium had a median (interquartile range) APACHE II score of 22 (17–28) compared with 14.5 (11–20) in non-delirium patients (*p* < 0.001). Likewise, SAPS II scores (45 (37–64) vs. 30 (23.5–42); *p* < 0.001) and SOFA scores (10 (5–13) vs. 4 (2–7); *p* < 0.001) were higher in patients with delirium. Admission mode was significantly different between the two groups (*p* < 0.001), with fewer surgical admissions (31% vs. 55%) and more emergency admissions (29% vs. 6%) in patients with delirium. Diagnose categories differed between the groups (*p* < 0.001), with fewer surgical patients among delirium patients (9% vs. 45%). Table A1 provides information on sedation levels and ventilation status on each day of assessment. 

### 3.2. Validity of Nu-DESC, ICDSC, and CAM-ICU across All Patients

**Figure 3:**
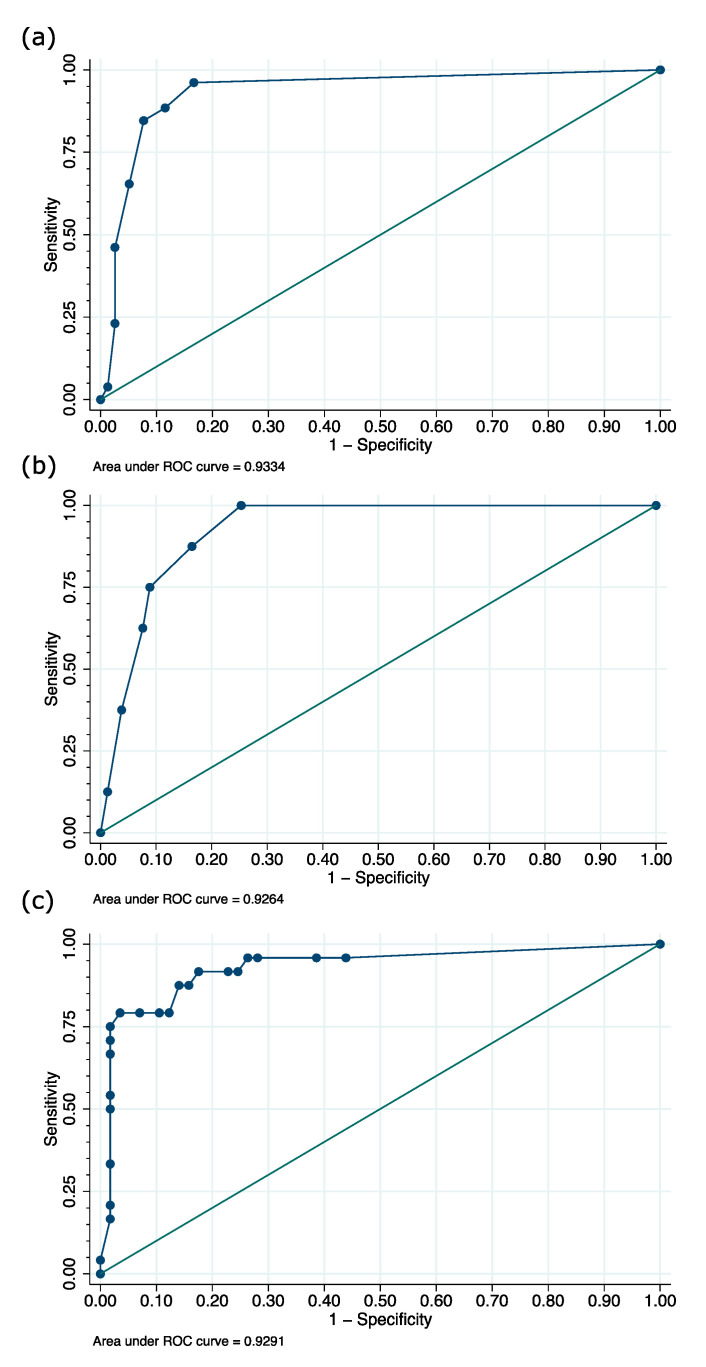
Receiver operating characteristic (ROC) curves for the scores of the (**a**) Nursing Delirium Screening Scale (Nu-DESC), (**b**) Intensive Care Delirium Screening Checklist (ICDSC), and (**c**) severity scale Confusion Assessment Method for the ICU (ssCAM-ICU).

In the first step, sensitivity, specificity, PPV, and NPV for each DSI were calculated in the study population (Table 2). The Nu-DESC revealed a sensitivity and a specificity of 88.5% in the population. In 47 patients, no delirium assessment with the Nu-DESC was performed because of deep sedation or missing data. The ICDSC revealed a sensitivity of 62.5% and a specificity of 92.4%. In 48 patients, delirium assessment with the ICDSC could not be performed due to deep sedation or missing data. The CAM-ICU revealed a sensitivity of 75.0% and a specificity of 94.7%. In 70 patients, delirium assessment with the CAM-ICU could not be performed due to deep sedation or missing data. Test validities of Nu-DESC and ICDSC (*p* = 0.004) as well as Nu-DESC and CAM-ICU (*p* = 0.008) were significantly different, but no significant difference was detected between ICDSC and CAM-ICU (*p* = 0.739). For Nu-DESC, ICDSC, and ssCAM-ICU, ROC analysis revealed an AUC of 0.93 for all scores (see Figure 3).

Taking into consideration the Youden index as well as individual sensitivity and specificity (both of at least 80%), the ideal cut-offs for the DSIs in our study population were 1 for the Nu-DESC (respective sensitivity 96.2% and specificity 83.3%) and 2 for the ICDSC (respective sensitivity 87.5% and specificity 83.5%). For the ssCAM-ICU, the ideal cut-off was determined as 5.5 points (respective sensitivity 91.7% and specificity 82.5%) to distinguish between delirious and non-delirious patients according to the neuropsychiatric assessment with DSM-IV-TR.

### 3.3. Validity of Delirium Assessment Using Nu-DESC, ICDSC, and CAM-ICU Depending on Sedation Level and Ventilation Status

Assessments were grouped according to patients’ sedation levels (RASS < −1; RASS 0/−1; RASS > 0) or ventilation status (no mechanical ventilation or mechanical ventilation). For each subgroup, sensitivity, specificity, PPV, and NPV were calculated (see Table A2). As indicated by the Youden index, test validities were highest in the non-sedated patient subgroup for all tests. For RASS 0/−1, the Nu-DESC had a sensitivity of 81.2% and specificity of 91.9%, the ICDSC a sensitivity of 62.5% and specificity of 95.4%, and the CAM-ICU a sensitivity of 72.2% and specificity of 100%. According to the Youden index, overall test validities were more problematic for sedated patients (RASS < −1), with particularly low specificities: the Nu-DESC had a sensitivity of 85.7% and a specificity of 0%, the ICDSC a sensitivity of 78.6% and a specificity of 25%, and the CAM-ICU a sensitivity of 86.7% and a specificity of 50%. For noninvasively ventilated patients, the Nu-DESC had a sensitivity of 87.5% and specificity of 87.7%, the ICDSC a sensitivity of 64.3% and specificity of 94.9%, and the CAM-ICU a sensitivity of 68.8% and specificity of 96.2%. Most patients belonged to the subgroup of RASS −1 or 0 (70–71% of patients) and noninvasively ventilated patients (79–82% of patients). 

**Figure 4:**
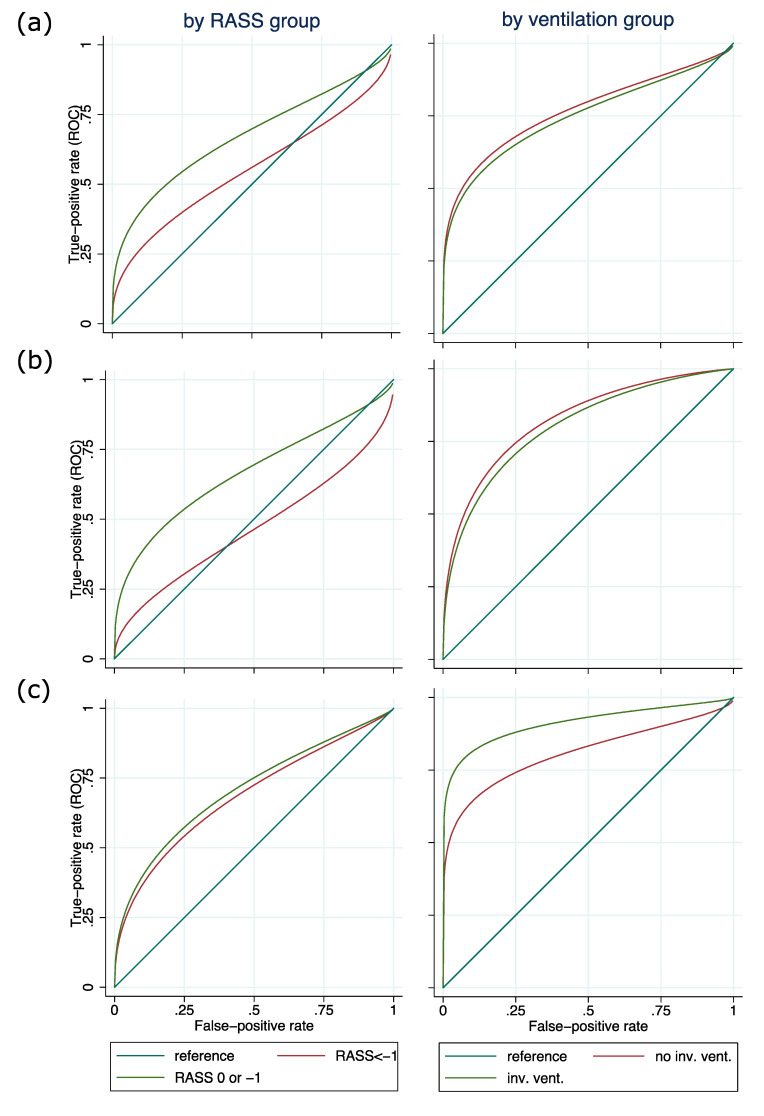
Receiver operating characteristic (ROC) regression analysis depending on level of sedation and ventilation status for (**a**) Nursing Delirium Screening Scale (Nu-DESC), (**b**) Intensive Care Delirium Screening Checklist (ICDSC), and (**c**) severity scale Confusion Assessment Method for the ICU (ssCAM-ICU). Plot of regression functions for deeply sedated patients and awake patients. Illustrating the results of the subgroup analysis for the Nu-DESC and the ICDSC, areas under the curve (AUCs) of the ROC curves of deeply sedated patients are smaller than AUCs for awake patients. For the ssCAM-ICU, there is little difference in AUC for deeply sedated and awake patients, indicating that the ssCAM-ICU keeps its reliability in deeply sedated patients. Likewise, the AUC of the ROC curves for invasively ventilated and noninvasively ventilated patients show little differences for Nu-DESC and ICDSC. For the ssCAM-ICU, however, the AUC for invasively ventilated patients is larger than it is for noninvasively ventilated patients. This might be interpreted as an indication that ssCAM-ICU is less affected by ventilation status than Nu-DESC and ICDSC. Abbreviations: RASS = Richmond Agitation Sedation Scale.

**Figure A1:**
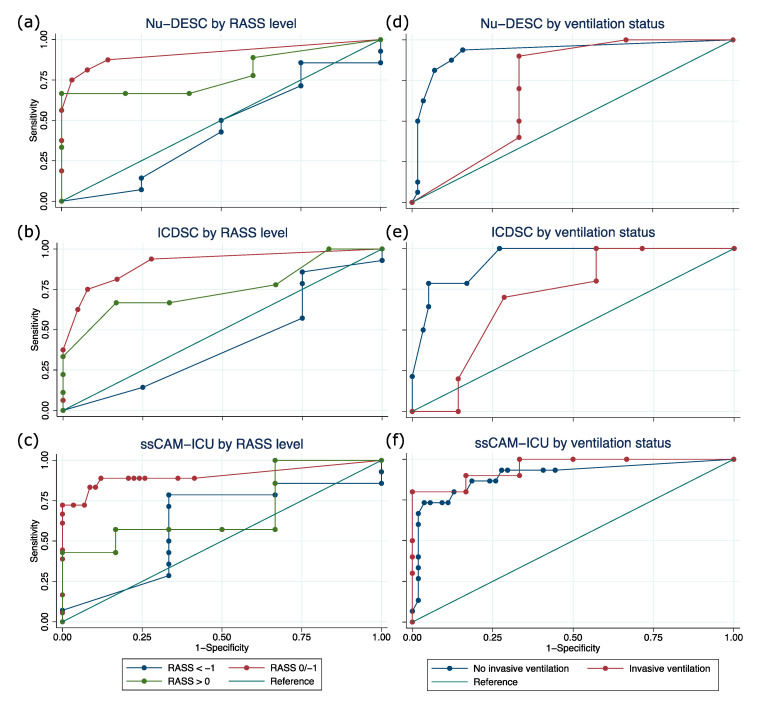
Receiver operating characteristic (ROC) curves by sedation level and ventilation status for the (**a**,**d**) Nursing Delirium Screening Scale (Nu-DESC), (**b**,**e**) Intensive Care Delirium Screening Checklist (ICDSC), and (**c**,**f**) severity scale Confusion Assessment Method for the ICU (ssCAM-ICU). (**a**) Area under the curve (AUC): Richmond Agitation Sedation Scale (RASS) < −1: 0.45 (n = 18); RASS 0/−1: 0.91 (n = 78); RASS > 0: 0.79 (n = 14). Chi2 (2) = 6.05, *p* = 0.049. (**b**) AUC: RASS < −1: 0.42 (n = 18); RASS 0/−1: 0.91 (n = 81); RASS > 0: 0.75 (n = 15). Chi2 (2) = 7.72, *p* = 0.021. **(c)** AUC: RASS < −1: 0.61 (n = 17); RASS 0/−1: 0.91 (n = 76); RASS > 0: 0.69 (n = 13). Chi2 (2) = 2.99, *p* = 0.225. (**d**) AUC: no invasive ventilation: 0.93 (n = 73); invasive ventilation: 0.72 (n = 16). Chi2 (1) = 1.50, *p* = 0.220. (**e**) AUC: no invasive ventilation: 0.93 (n = 73); invasive ventilation: 0.71 (n = 17). Chi2 (1) = 2.22, *p* = 0.136. (**f**) AUC: no invasive ventilation: 0.90 (n = 69); invasive ventilation: 0.95 (n = 16). Chi2 (1) = 0.45, *p* = 0.502.

In the next step, ROC curves for each subgroup were plotted and AUC was determined (Figure A1). For sedation level subgroups, AUC was greatest for patients with RASS 0/−1 across all test instruments (Nu-DESC, ICDSC, and ssCAM-ICU: 0.91). AUCs of sedation level subgroups differed significantly for Nu-DESC (*p* = 0.049) and ICDSC (*p* = 0.021), whereas for the ssCAM-ICU, AUCs were not significantly different (*p* = 0.225). For ventilation status subgroups, AUC was larger in patients without invasive ventilation for Nu-DESC (0.72 vs. 0.93, *p* = 0.220) and ICDSC (0.71 vs. 0.93, *p* = 0.136). For ssCAM-ICU, however, AUC showed a nonsignificant trend to be larger for patients being invasively ventilated (0.95 vs. 0.90, *p* = 0.502). The results of the regression analysis, adjusting ROC curves for sedation levels and ventilation status, can be found in Figure 4.

## 4. Discussion

This observational study examined the test validity of different DSIs with regard to sedation and ventilator status in critically ill adult patients. We were able to show that test validity was best with awake or drowsy patients (RASS 0/−1) for all tests. However, differences in test validity between different levels of sedation were only significant in those DSIs relying on observations (ICDSC and Nu-DESC) and not the ssCAM-ICU, which showed a nonsignificant trend towards a better performance in patients with RASS 0/−1 compared with patients with RASS < −1. There were no significant differences in test validities in terms of ROC-AUC between ventilated and nonventilated patients.

The global sensitivities and specificities of the DSIs were comparable to those in previous studies that used neuropsychological examination as the reference standard in the critical care context [7,9,13,16,20,21,22,23]. CAM-ICU and ICDSC showed excellent specificity (CAM-ICU: 94.7% and ICDSC: 92.4%) and moderate-to-good sensitivity (CAM-ICU: 75% and ICDSC: 62.5%), whereas the Nu-DESC showed the best sensitivity (88.5%) with an overall slightly lower specificity (88.5%). However, test sensitivities show a broad range between different studies, ranging from 98% for CAM-ICU and 99% for the ICDSC to 64% and 43%, respectively [13]. Various factors might contribute to this difference, including the prevalence of delirium in the studied population that varies from more than 80% to 16%, the way the neuropsychological testing is conducted, as well as the test itself. Meagher et al., for example, found that there is considerable variation in patients being classified as delirious after a neuropsychological assessment by the DSM-5 as a reference standard, depending on the use of a “strong” or “relaxed” interpretation of the criteria [24]. We used the DSM-IV-TR criteria, which were also used in comparable studies, in a very standardized and comprehensive manner and excluded patients that were deeply sedated from the assessment. Thus, the investigator-related factors were largely taken into account. Also, the delirium incidence in our study cohort was in line with that of previous investigations in comparable populations, which impacts results as discussed by Neufeld and colleagues in the noncritical care context [25]. When considering differences in test performances between studies for a particular DSI, aspects of sufficient staff training play an important role as well. Training requirements are different for each of the scores. CAM-ICU and ICDSC are known to require an extensive training, whereas literature suggests that the Nu-DESC requires less training and shows very good validity in recovery rooms and peripheral wards [26]. In our study, all examiners were extensively trained in the use of their respective score to account for training effects, but this might explain variations in test validities between studies.

An important finding of our study is that the ideal cut-offs of the observational scales in our setting were 1 (instead of 2) for the Nu-DESC and 2 (instead of 4) for the ICDSC. There have been other studies discussing whether a lower cut-off for the ICDSC might be beneficial [27,28]. Our data is in line with these studies, indicating that a lower threshold could increase the diagnostic performance. This has also been shown for other delirium scores like the Nu-DESC and the Delirium Detection Score, where lower cut-offs have increased sensitivity [26]. Whether individual thresholds for certain subgroups are feasible should be investigated in future studies, as our subgroup sizes did not have sufficient statistical power for this analysis.

No study to date had investigated the influence of covariates on sensitivity and specificity of DSIs in the adult critical care population. In the pediatric critical care field, one observational study assessed the effect of covariates (age, gender, and sedation) on the diagnostic performance of pediatric delirium scales [16]. That study demonstrated that RASS levels had a significant effect on corresponding ROC curves of the tested DSIs, which is per se in line with our results. In contrast to our findings, the sspCAM-ICU showed a significantly better performance in awake patients than in deeply sedated patients in the pediatric population. A possible explanation is that in deeply sedated patients, DSIs are more likely to be judged positively in the awareness domains, leading to an overall positive result, although the patient is not delirious but only sedated. This has been referred to as “sedation-induced, rapid-reversible delirium” [29]. Although sedation might mimic some clinical features of delirium, it does not resemble delirium from a pathophysiological point of view. If delirium ceases with termination of sedation, these patients do not show the same impaired outcome [29]. In the DSM-5, delirium is defined more restrictively and the term consciousness itself is no longer part of the definition. It is specified that inattention and changes in cognition “should not occur in the context of a severely reduced level of arousal such as coma”. Although this might lead to a rather restrictive application [30], which potentially leads to an underdiagnosis of delirium, the nonexclusion of sedation might lead to an overdiagnosis when using DSIs. Another aspect to consider when interpreting the influence of sedation on DSI performance is the fact that sedated, delirious patients (e.g., RASS −2) may show alterations in the composition of their delirium symptoms, for example, patients might show less agitation and less fluctuation. This might have a negative impact on the outcome of the delirium screening instruments themselves, explaining the significantly worse performance of DSIs in delirious patients.

We could not show any significant effect of mechanical ventilation on performance of the DSIs. However, this might be due to the small size of the subgroup of mechanically ventilated patients and requires further scrutiny in the future. As for now, there is no sufficient evidence to prefer one DSI over another for ventilated patients in the critical care context. Nevertheless, it should be mentioned that other studies with nonventilated patients usually showed lower diagnostic validities, which have been attributed to the fact that the patients were not ventilated [25,31].

When interpreting the results of our study, limitations have to be taken into consideration. We used a neuropsychiatric examination as the reference standard in the critical care population. On the one hand, delirium in and outside the ICU has the same definition, and, thus, should be diagnosed equally according to DSM. On the other hand, the application of a neuropsychiatric examination is more difficult in ICU patients as ventilation, medication, and—especially in highly acute settings—incomplete medical history can impede judgement [32]. We mitigated this limitation by using a structured approach and training for all assessors to standardize judgement. Clinical routine and quality management data prior to the study revealed an excellent inter-rater reliability in 15 assessments (not shown; k > 0.9). In ambiguous cases, the examiners were encouraged to consult a senior specialist to decide on delirium diagnosis together. It also needs to be mentioned that we used the DSM-IV-TR instead of the DSM-5 criteria. At the time of data acquisition, DSM-IV-TR was the standard of reference. Further, as the application of the DSM-5 criteria would significantly decrease comparability to other studies that used previous versions of the DSM, it seems rational to use the DSM-IV-TR. Another limitation of this study concerns the state of sedation in patients enrolled in the study. As our assessment took between 30 min and one hour, sedation holds in patients receiving deep sedation for a special indication could not be performed. This is a limitation, because the DSIs might perform differently during sedation holidays. Particularly, nonstimulating, observational tests have a different performance. However, as the study sites followed a sedation protocol targeting no or light sedation (target RASS 0/−1), the majority of patients was seen in a stable sedative state. Furthermore, the unequal subgroup sizes in our patient cohort impose limitations on statistical power and interpretation. For the sample size calculation ahead of data acquisition, we assumed equal subgroup sizes and did not account for skewed subgroup distributions. Future studies should take this into account and increase the sample size to avoid small subgroups.

## 5. Conclusions

In summary, our international, bi-center, prospective observational study shows that sedation has a statistically significant effect on the diagnostic validity of the observational DSIs ICDSC and Nu-DESC. Practitioners using these scores should therefore consider sedation as a cause for a flawed delirium screening and refrain from sedation whenever clinically reasonable. Although the (ss)CAM-ICU showed similar trends, the differences between levels of sedation did not reach significance. The score can therefore be considered more robust regarding these variables.

## Figures and Tables

**Table 1:** Descriptive statistics of the study population.

	No Delirium (n = 116)	Delirium * (n = 35)	*p*
Age, yr	67 (53–75) ^a^	68 (50–74) ^a^	0.824 ^b^
Height, cm	170 (163–175) ^a^	168 (160–177) ^a^	0.702 ^b^
Weight, kg	75 (65–85) ^a^	73 (65–80) ^a^	0.415 ^b^
BMI, kg/m^2^	25 (23–29) ^a^	25 (23–29) ^a^	0.784 ^b^
APACHE II score	14.5 (11–20) ^a^	22 (17–28) ^a^	<0.001 ^b^
SAPS II score	30 (23.5–42) ^a^	45 (37–64) ^a^	<0.001 ^b^
SOFA score	4 (2–7) ^a^	10 (5–13) ^a^	<0.001 ^b^
Male, n	61	17	0.703 ^c^
Admission mode, n			
Emergency	7	10	<0.001 ^d^
Medical	45	14	
Surgical	64	11	
Diagnose group, n			
Acute respiratory failure	33	13	<0.001 ^d^
Surgical, postoperative	52	3	
Trauma, bleeding	10	10	
Others	21	9	

* Delirium on at least one occasion during assessment period, ^a^ data presented as median (limits of the interquartile range), ^b^ Mann–Whitney U test, ^c^ Fisher’s exact test, ^d^ Chi-squared test. Abbreviations: BMI = Body mass index; APACHE II = Acute Physiology and Chronic Health Disease Classification System II; SAPS II = Simplified Acute Physiology Score II.

**Table 2:** Sensitivity, specificity, and positive as well as negative predictive values for the Nursing Delirium Screening Scale (Nu-DESC), the Intensive Care Delirium Screening Checklist (ICDSC), and the Confusion Assessment Method for the ICU (CAM-ICU). Abbreviations: DSI = Delirium screening instrument; CI = Confidence interval.

DSI	n	Sensitivity		Specificity		Positive Predictive Value		Negative Predictive Value	
		Estimate (%)	CI (%)	Estimate (%)	CI (%)	Estimate (%)	CI (%)	Estimate (%)	CI (%)
Nu-DESC ^1,2^	104	88.5	69.8–97.6	88.5	79.2–94.6	71.9	53.3–86.3	95.8	88.3–99.1
ICDSC ^1,3^	103	62.5	40.6–81.2	92.4	84.2–97.2	71.4	47.8–88.7	89.0	80.2–94.9
CAM-ICU ^2,3^	81	75.0	53.3–90.2	94.7	85.4–98.9	85.7	63.7–97.0	90.0	79.5–96.2

^1^ Nu-DESC vs. ICDSC: *p* = 0.004; ^2^ Nu-DESC vs. CAM-ICU: *p* = 0.008; ^3^ ICDSC vs. CAM-ICU: *p* = 0.739 (McNemar test).

## Appendix A

**Table A1:** Prevalence of delirium, sedation levels, and ventilation status by day of assessment. Abbreviations: RASS = Richmond Agitation Sedation Scale.

Day		1		2		3		4		5		6		7	
		n	%	n	%	n	%	n	%	n	%	n	%	n	%
Delirium	Yes	22	14.6	14	14.3	15	22.4	15	28.3	12	27.9	11	30.6	5	15.6
	No	73	48.3	39	39.8	33	49.3	23	43.4	20	46.5	16	44.4	13	40.6
	RASS < –3	51	33.8	30	30.6	14	20.9	9	17.0	7	16.3	7	19.4	6	18.8
	No data	5	3.3	15	15.3	5	7.5	6	11.3	4	9.3	2	5.6	8	25.0
RASS group	<–1	35	23.2	19	19.4	21	31.3	18	34.0	15	34.9	11	30.6	10	31.3
	–1/0	91	60.3	60	61.2	36	53.7	24	45.3	20	46.5	19	52.8	19	59.4
	>0	8	5.3	11	11.2	4	6.0	5	9.4	4	9.3	3	8.3	3	9.4
	No data	17	11.3	8	8.2	6	9.0	6	11.3	4	9.3	3	8.3	0	0.0
Invasive ventilation	Yes	40	26.5	23	23.5	24	35.8	20	37.7	17	39.5	15	41.7	17	53.1
	No	92	60.9	63	64.3	39	58.2	27	50.9	23	53.5	18	50.0	13	40.6
	No data	19	12.6	12	12.2	4	6.0	6	11.3	3	7.0	3	8.3	2	6.3
Total		151	100.0	98	100.0	67	100.0	53	100.0	43	100.0	36	100.0	32	100.0

**Table A2:** Test validity for the Nursing Delirium Screening Scale (Nu-DESC), Intensive Care Delirium Screening Checklist (ICDSC), and Confusion Assessment Method for the ICU (CAM-ICU) by sedation level and ventilation status. Abbreviations: DSI = Delirium screening instrument; CI = Confidence interval; RASS = Richmond Agitation Sedation Scale; Inv. Vent. = Invasive ventilation.

DSI	Subgroup		Sensitivity		Specificity		Positive Predictive Value		Negative Predictive Value		Youden Index
		n	Estimate (%)	CI (%)	Estimate (%)	CI (%)	Estimate (%)	CI (%)	Estimate (%)	CI (%)	
Nu-DESC	RASS <−1	18	85.7	57.2–98.2	0.0	0.0–60.2	75.0	47.6–92.7	0.0	0.0–84.2	−0.14
	RASS −1/0	78	81.2	54.4–96.0	91.9	82.2–97.3	72.2	46.5–90.3	95.0	86.1–99.0	0.73
	RASS > 0	14	77.8	40.0–97.2	40.0	5.3–85.3	70.0	34.8–93.3	50.0	6.8–93.2	0.18
	Inv. Vent.	16	90.0	55.5–99.7	66.7	22.3–95.7	81.8	48.2–97.7	80.0	28.4–99.5	0.57
	No Inv. Vent.	73	87.5	61.7–98.4	87.7	76.3–94.9	66.7	43.0–85.4	96.2	86.8–99.5	0.75
ICDSC	RASS <−1	18	78.6	49.2–95.3	25.0	0.6–80.6	78.6	49.2–95.3	25.0	0.6–80.6	0.04
	RASS −1/0	81	62.5	35.4–84.8	95.4	87.1–99.0	76.9	46.2–95.0	91.2	81.8–96.7	0.58
	RASS > 0	15	66.7	29.9–92.5	83.3	35.9–99.6	85.7	42.1–99.6	62.5	24.5–91.5	0.50
	Inv. Vent.	17	70.0	34.8–93.3	71.4	29.0–96.3	77.8	40.0–97.2	62.5	24.5–91.5	0.41
	No Inv. Vent.	73	64.3	35.1–87.2	94.9	85.9–98.9	75.0	42.8–94.5	91.8	81.9–97.3	0.59
CAM-ICU	RASS < −1	19	86.7	59.5–98.3	50.0	6.8–93.2	86.7	59.5–98.3	50.0	6.8–93.2	0.37
	RASS −1/0	76	72.2	46.5–90.3	100.0	93.8–100.0	100.0	75.3–100	92.1	82.4–97.4	0.72
	RASS > 0	14	62.5	24.5–91.5	50.0	11.8–88.2	62.5	24.5–91.5	50.0	11.8–88.2	0.13
	Inv. Vent.	18	90.9	58.7–99.8	71.4	29.0–96.3	83.3	51.6–97.9	83.3	35.9–99.6	0.62
	No Inv. Vent.	69	68.8	41.3–89.0	96.2	87.0–99.5	84.6	54.6–98.1	91.1	80.4–97.0	0.65
